# Effect of Auxin on Cadmium Toxicity-Induced Growth Inhibition in *Solanum lycopersicum*

**DOI:** 10.3390/toxics12050374

**Published:** 2024-05-19

**Authors:** Huabin Liu, Yue Wu, Jiahui Cai, Yuting Chen, Cheng Zhou, Cece Qiao, Yuliang Wang, Song Wang

**Affiliations:** 1 College of Life and Health Sciences, Anhui Science and Technology University, Chuzhou 233100, China; liuhuabin@mail.nankai.edu.cn (H.L.); wy16605662883@126.com (Y.W.); caijh@ahstu.edu.cn (J.C.); zhoucheng@njau.edu.cn (C.Z.); 15150535579@163.com (C.Q.); 2 College of Life Sciences, Nanjing Agricultural University, Nanjing 210095, China; 2023816122@stu.njau.edu.cn

**Keywords:** tomato, auxin, yucasin, root growth, Cd tolerance

## Abstract

Auxins play crucial regulatory roles in plants coping with cadmium (Cd) stress. However, the regulatory mechanism by which auxins alleviate Cd toxicity in tomato seedlings remains unclear. Here, we demonstrate that exposure to Cd stress leads to dynamic changes in the auxin response in tomato roots, characterized by an initial increase followed by a subsequent weakening. Under Cd stress, tomato seedlings show primary root- and hypocotyl-growth inhibition, accompanied by the accumulation of Cd and reactive oxygen species (ROS) in the roots. The exogenous application of 1-naphthylacetic acid (NAA) does not mitigate the inhibitory effect of Cd toxicity on primary root growth, but it does significantly enhance lateral root development under Cd stress. Auxin transport inhibitors, such as 1-N-naphthylphthalamic acid (NPA) and 2,3,5-triiodobenoic acid (TIBA), aggravate the growth inhibition of primary roots caused by Cd stress. Additionally, lateral root development was inhibited by NPA. However, applying auxin synthesis inhibitors L-kynurenine (kyn) and yucasin alleviated the tomato root growth inhibition caused by Cd stress; between them, the effect of yucasin was more pronounced. Yucasin mitigates Cd toxicity in tomato seedlings by reducing Cd^2+^ absorption and auxin accumulation, strengthening ROS scavenging, and reducing cell death in roots. These observations suggest that yucasin potentially mitigates Cd toxicity and improves the tolerance of tomato seedlings to Cd stress.

## 1. Introduction

Heavy metal pollution is a serious environmental challenge for the survival of plants and animals, with cadmium (Cd) posing the most severe threat to plant growth and human health. With recent increasing anthropogenic Cd emissions, such as through industrial production, mining, and extensive pesticide and chemical fertilizer application, Cd soil contamination has become a prominent issue [1,2]. Cd exhibits strong migration characteristics. The roots easily absorb Cd^2+^ from the soil, which accumulates in plants and causes phytotoxicity [3,4]. Cd accumulation in the food chain eventually threatens human health. Tomatoes are an economically important and widely cultivated crop; however, they are often subjected to environmental stress during cultivation. Tomato development and quality are often influenced by Cd stress [5,6]. Thus, it is imperative to reveal the regulatory mechanism underlying the response of tomatoes to Cd stress in order to enhance Cd tolerance and breed Cd-tolerant tomato varieties.

During long-term evolution, plants have developed multiple adaptations and detoxification strategies to cope with Cd toxicity. Adaptations include reducing Cd absorption, enhancing Cd efflux from the roots, and regulating Cd distribution [7]. Detoxification mechanisms include producing chelatins to chelate Cd^2+^ and form a nontoxic chelate, improving the cell wall’s ability to bind and retain Cd^2+^ to reduce Cd entry into the protoplast, vacuole compartmentalization to sequester Cd, and enhancing the antioxidant capacity of the cell [7,8]. Nonetheless, excess Cd^2+^ accumulation can irreversibly damage plant growth, most evidently by inhibiting root growth [9]. Cd stress can also reduce the chlorophyll content of plants, affect key photosynthetic enzyme activity, and destroy the photosynthetic system, thereby reducing photosynthetic efficiency [10,11].

ROS, as a crucial stress response signaling molecule, play vital roles in plants coping with Cd stress [12]. However, excessive ROS accumulation due to Cd stress can cause secondary damage to plant cells, including oxidative damage to the plasma membrane system, destroying cellular activities, and inducing programmed cell death [13]. Scavenging Cd-induced ROS can effectively reduce Cd toxicity in plants [14]. Exogenous selenium application can reduce Cd absorption, enhance antioxidant capacity, and improve tobacco resistance to Cd [15]. However, there is still a lack of research on identifying effective exogenous substances that can improve tomato Cd stress tolerance.

The cell walls are the natural obstacle to Cd^2+^ migration into the cytoplasm and are key for reducing Cd toxicity in plants [14,16]. However, the cell wall may also be affected by Cd [16]. The components of the cell wall are cellulose, hemicellulose, and pectin. Both pectin and hemicellulose can bind to Cd^2+^ [16,17]. The hemicellulose and pectin contents are positively correlated with Cd^2+^ accumulation [18,19]. However, whether the combination of cell wall polysaccharides (hemicellulose and pectin) improves plant resistance to Cd remains controversial. A study shows that reducing cell wall pectin levels leads to reduced Cd accumulation, thereby alleviating Cd-induced root growth inhibition [20]. In contrast, auxin analog NAA application enhances hemicellulose 1 content, facilitates Cd ion sequestration in the root cell wall of Arabidopsis, and restricts Cd^2+^ transport from roots to stems, thereby mitigating aboveground Cd accumulation and reducing its phytotoxicity [19]. In addition, recent research reported that exogenous NAA can also reduce Cd accumulation by reducing hemicellulose contents in rice [21]. These conflicting experimental results imply a complex auxin regulation of plant growth during Cd stress.

Auxins are important plant hormones involved in regulating plant stress responses. The study has demonstrated that auxin levels and homeostasis in plants are affected by Cd stress [9]; however, conflicting evidence supports inconsistent or opposing auxin response patterns during Cd stress. Li et al. [21] reported that YUCCAs expression is upregulated under Cd stress, accompanied by increased endogenous auxin levels in rice. However, other studies suggest that auxin accumulation is reduced in the root under Cd stress [15,19]. These findings imply that there is a complex auxin signaling response in plants under Cd stress. In Arabidopsis, auxin transport inhibitor NPA application has no impact on primary root elongation but does suppress Cd-induced lateral root formation [9]. Consistent with this, the primary roots of *aux1* and *pin2*, auxin transport-related mutants, are consistent with the wild type and exhibited sensitivity to Cd, while lateral root development in *aux1* and *pin2* differed from that of the wild type and showed insensitivity to Cd stress [9]. NAA application could effectively alleviate the toxicity of Cd stress to Arabidopsis [19]. Despite this, research on Cd stress in tomatoes is still limited; in particular, there are few reports on plant growth regulator applications such as auxins to alleviate Cd toxicity in tomatoes. Although numerous studies have reported that auxins play important regulatory roles in plant coping with Cd stress, plant defense mechanisms remain poorly understood [22]. In particular, the screening of effective exogenous chemicals to address the issue of plant cadmium stress in agricultural production will offer substantial support for ensuring safe agricultural practices.

Our findings indicate that exposure to Cd stress induces an auxin response in tomato roots with a dynamic distribution depending on different exogenous Cd concentrations and treatment durations. This study aimed to identify effective methods to address plant Cd toxicity through auxin synthesis, transport, and signaling pathways. Exogenous auxin application can partially alleviate hypocotyl elongation inhibition and promote lateral root development in tomato seedlings. Treatment with the auxin synthesis inhibitor yucasin significantly stimulated primary root growth and reduced Cd toxicity. Overall, our research aimed to reveal the regulatory mechanism of auxins in tomato root growth exposed to Cd stress.

## 2. Materials and Methods

### 2.1. Seedling Cultivation

Tomatoes (*Solanum lycopersicum* cv. Aisheng) were selected for our experiments. A Micro-Tom expressing *DR5:GUS* auxin-responsive reporter was used for DR5 signal analysis. For seedling culture, tomato seeds were placed in 1/2 MS medium for germination at 24 °C in darkness. The tomato seedlings were further cultured vertically for five days under a 14 h photoperiod. Subsequently, tomato seedlings were kept in a 1/5 Hoagland solution for 12 h pretreatment before being subjected to different experimental treatments.

### 2.2. CdCl_2_ Treatment

For CdCl_2_ treatment, the tomato seedlings were transferred to a nutrient solution containing different concentrations of CdCl_2_. The culture solution was refreshed every two days.

### 2.3. Hormone Treatments

For root growth experiments, five-day-old tomato seedlings were incubated in the nutrient solution containing 25 μM CdCl_2_ or CdCl_2_ plus various concentrations of NAA (2.5–50 nM), IAA (2.5–50 nM), kyn (2.5–20 μM), yucasin (2.5–20 μM), TIBA (0.5–4 μM), or NPA (1–8 μM) for six days. The solution was refreshed every 2 days. Tomato seedlings were photographed using a scanner (Epson Perfection V33) at 0, 2, 4, and 6 days after treatment, and the root and hypocotyl lengths were measured using the ImageJ software package. IAA (Sangon) was dissolved in 95% ethanol, and NAA (Sangon), NPA (Sigma), TIBA (Sangon), kyn (Macklin), and yucasin (Aladdin) were dissolved in DMSO.

### 2.4. GUS Staining

To detect *DR5:GUS* expression, tomato seedlings were subjected to 25 μM CdCl_2_ for indicated times, then immersed in GUS solution for 12 h at 37 °C, as described [23]. Subsequently, tomato seedlings were cleared in a solution [23] and observed under a microscope.

### 2.5. ROS and Cell Death Detection

ROS were detected using diaminobenzidine (DAB) staining [24]. Five-day-old seedlings were subjected to CdCl_2_ or CdCl_2_ plus yucasin for two hours. Subsequently, samples were dipped in 1 mg/mL DAB solution [25] for 30 min, rinsed with ddH_2_O for three min, and observed using a microscope.

Cell death was monitored by trypan blue staining [26]. Five-day-old seedlings were subjected to CdCl_2_ or CdCl_2_ plus yucasin for 12 h. Subsequently, the samples were dipped in trypan blue solution [25] for 15 min, rinsed with ddH_2_O for 5 min, and observed using a microscope.

### 2.6. Cd Content Analysis

Tomato seedlings were placed in the nutrient solution containing CdCl_2_, IAA, or CdCl_2_ with IAA or yucasin for four days. The solution was refreshed every two days.

For Cd content analysis, the tomato seedling roots and aboveground parts were collected and dried. Grind the dry plant tissue (0.5 g) into powder and digest with 65% HNO_3_. Then, the samples were pretreated by adding 5 mL of HNO_3_ for 12 h, digested at 120 °C for three hours. After cooling, we added 1 mL H_2_O_2_ to the solution until it became transparent. Cd level was determined using ICP–MS [12].

### 2.7. Photosynthetic Pigment Measurement

To quantify the photosynthetic pigments, the chlorophyll and carotenoid contents were measured as described previously [27]. The leaves were ground into a powder and dissolved in 2 mL of 95% ethanol. The absorption wavelengths for detecting chlorophyll a, b, and carotenoids were 665, 649, and 470 nm, respectively.

### 2.8. Statistical Analysis

Data analysis was performed using a Student’s *t*-test or ANOVA (Duncan’s test). Each experiment was conducted with 3–5 biological replicates and 8–15 tomato seedlings per treatment. Statistical significance was defined as *p* < 0.05.

## 3. Results

### 3.1. Cd stress Inhibits Tomato Seedling Growth

**Figure 1:**
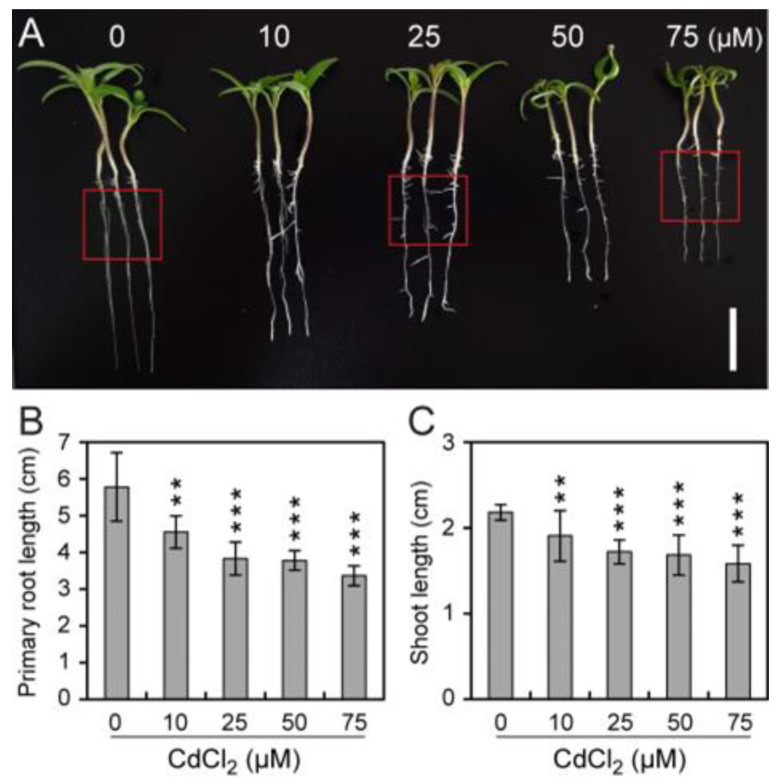
Impact of CdCl_2_ on tomato root and hypocotyl growth. (**A**,**B**). Tomato seedlings were transferred to a hydroponic treatment solution supplemented with 10, 25, 50, or 75 μM CdCl_2_ for six days. The tomato seedling phenotype (**A**), root length (**B**), and hypocotyl length (**C**) (*n* ≥ 8, average ± SD) were compared using the Student’s *t*-test (** *p* < 0.01, *** *p* < 0.001). Red boxes indicate lateral roots. Scale bar = 2 cm.

The impacts of Cd toxicity on plant growth were assessed by treating tomato seedlings with various concentrations of CdCl_2_. Tomato seedlings exposed to CdCl_2_ exhibited significantly reduced root and hypocotyl growth. As the CdCl_2_ concentration increased, seedling growth was significantly inhibited (Figure 1 and Figure S1). Compared with the controls, tomato seedlings exhibited 21.2% and 12.7% reductions in root and hypocotyl length, respectively, following treatment with 10 μM CdCl_2_ (Figure 1B,C). Similarly, 25 μM CdCl_2_ treatment decreased root and hypocotyl length by 33.7% and 21.2%, respectively (Figure 1B,C), while the number of lateral roots significantly increased (Figure S1A). Additionally, 75 μM CdCl_2_ treatment decreased root and hypocotyl length by 41.9% and 27.4%, respectively (Figure 1B,C). We also found that there was a noticeable reduction in the lateral roots after CdCl_2_ treatment (Figure S1A). These findings indicate that Cd inhibits tomato seedling growth, including primary root and hypocotyl elongation. In addition, low concentrations of Cd^2+^ promote lateral root development, while high concentrations of Cd^2+^ inhibit lateral root development.

### 3.2. Auxin Is Involved in the Response of Tomato Seedlings to Cd Stress

**Figure 2:**
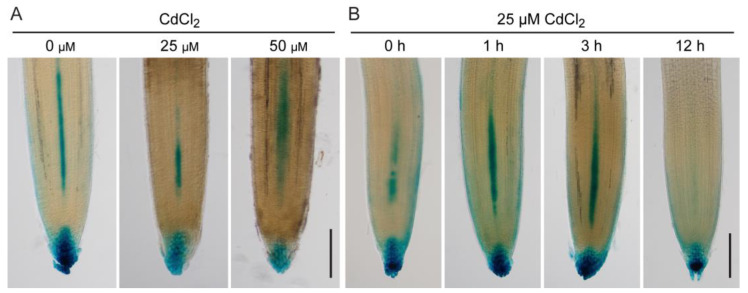
Changes in auxin accumulation and distribution induced by Cd stress in roots. (**A**). Five-day-old seedlings (*DR5:GUS*) were subjected or not to CdCl_2_ (25 and 50 μM) for six hours. Scale bar = 200 μm. (**B**). Tomato seedlings (*DR5:GUS*) were subjected to CdCl_2_ (25 μM) for the indicated times. Scale bar = 200 μm.

Previous research has demonstrated that auxin is responsible for regulating root growth under Cd stress [12,13,15,19]. However, conflicting evidence supports the involvement of auxin in plant responses to Cd toxicity [28,29]. To elucidate the mechanism of auxin in tomato seedlings response to Cd stress, the *DR5:GUS* reporter gene was used to monitor the distribution pattern of auxin in tomato roots. The results show that the auxin response signal was attenuated in both the root tip and stele of seedlings after 25 μM CdCl_2_ treatment for six hours (Figure 2A). Moreover, exposure to 50 μM CdCl_2_ for six hours resulted in the diffusion of the signal within the stele (Figure 2A). Time-series analysis revealed that exposure to 25 μM CdCl_2_ for one or three hours resulted in enhanced auxin signals in the root tip, meristem, and stele (Figure 2B). This implies that Cd-induced root growth inhibition might be related to the transient accumulation of auxin in response to Cd. There was an unexpected, significant attenuation in the auxin response in the root tip and stele after 12 h of exposure to CdCl_2_ stress (Figure 2B). These findings imply that auxin serves as a response signal to Cd toxicity and regulates root growth.

### 3.3. Impact of Auxin on Tomato Root Growth under Cd Stress

**Figure 3:**
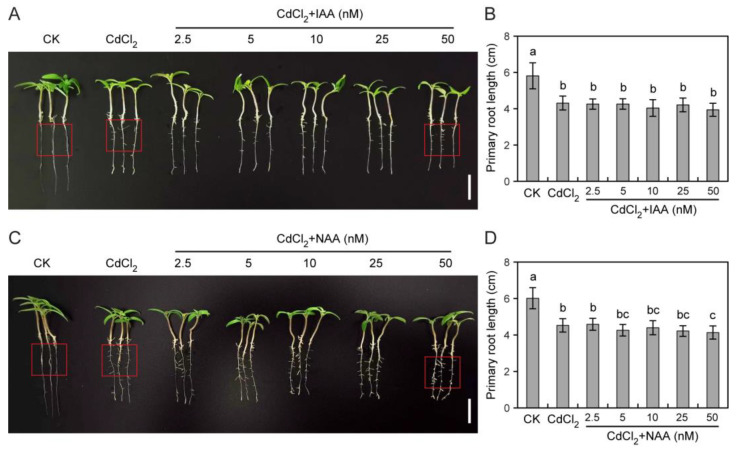
Effects of exogenous auxins on tomato seedling growth under CdCl_2_ treatment. (**A**–**D**). Effects of IAA and NAA on Cd-induced root inhibition in tomatoes. Five-day-old seedlings were subjected to 25 μM CdCl_2_ or 25 μM CdCl_2_ with 2.5–50 nM IAA (**A**,**B**) or NAA (**C**,**D**) for six days. Values represent the average ± SD (*n* ≥ 8, Duncan’s test). Red boxes indicate lateral roots. Scale bar = 2 cm.

To elucidate the actions of auxins on tomato seedling growth under Cd stress, the regulatory mechanism of auxins was investigated. First, we examined the effects of the exogenous auxin IAA and the auxin analog NAA on seedling growth under Cd stress. As shown in Figure 3A, exogenous IAA application did not mitigate tomato root growth inhibition under Cd stress. However, 10 nM IAA ameliorated hypocotyl growth in tomato seedlings (Figure S2A). IAA had no obvious effect on lateral tomato root development under Cd stress (Figure 3A,B and Figure S3B). Similarly, NAA did not mitigate the impact of Cd toxicity on tomato root elongation (Figure 3C,D and Figure S4). High-concentration NAA treatment aggravated Cd-induced root growth inhibition (Figure 3C,D). However, NAA significantly promoted lateral root development under Cd stress (Figure 3C and Figure S3A). In addition, 2.5 nM NAA mitigated hypocotyl growth inhibition caused by CdCl_2_ treatment (Figure S2B).

**Figure 4:**
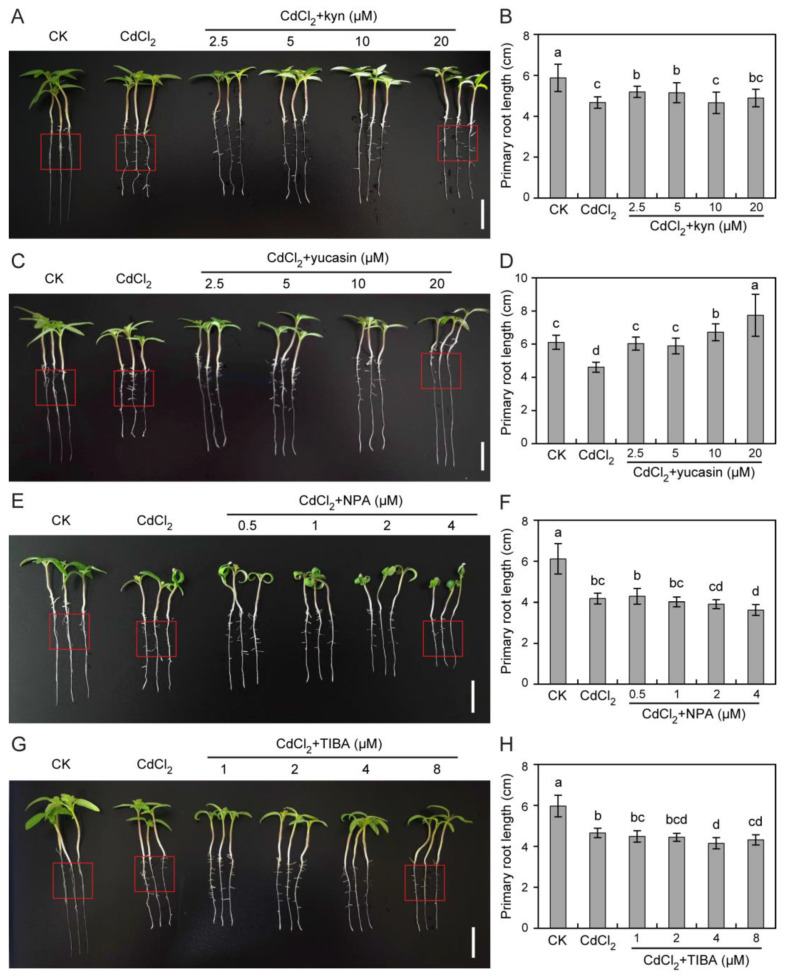
Effects of auxin inhibitors on tomato root growth under CdCl_2_ treatment. (**A**–**H**). Effects of auxin biosynthesis inhibitors (kyn and yucasin) and transport inhibitors (NPA and TIBA) on Cd-induced tomato root inhibition in tomatoes. Five-day-old tomato seedlings were subjected to 25 μM CdCl_2_, or 25 μM CdCl_2_ with 2.5–20 μM kyn (**A**,**B**) or yucasin (**C**,**D**), or 0.5–4 μM NPA (**E**,**F**), or 1–8 μM TIBA (**G**,**H**) for 6 days. Values represent the average ± SD (*n* ≥ 8, Duncan’s test). Red boxes indicate lateral roots. Scale bar = 2 cm.

To assess the influence of endogenous auxin on tomato seedling growth under Cd stress, auxin levels were manipulated via auxin synthesis inhibitor application using kyn [30] and yucasin [31]. We found that 2.5 or 5 μM kyn partially alleviated Cd-induced root growth inhibition (Figure 4A,B); however, it failed to mitigate the inhibitory effect of Cd on hypocotyl elongation (Figure S2C). Surprisingly, yucasin promoted tomato root elongation under Cd stress (Figure 4C,D and Figure S4). Compared with CdCl_2_ treatment, 2.5, 5, 10, and 20 μM yucasin treatment increased root length by 30.8%, 27.8%, 45.7%, and 77.3%, respectively, in the presence of 25 μM CdCl_2_ (Figure 4D). However, the number of lateral roots in yucasin-treated seedlings was significantly reduced (Figure 4C and Figure S3D). Yucasin did not mitigate hypocotyl growth inhibition caused by Cd toxicity (Figure S2D). These findings suggest that yucasin application could effectively alleviate Cd-induced primary root inhibition.

Subsequently, we explored whether polar auxin transport (PAT) was also involved in tomato seedling growth under Cd stress via exogenous application of auxin transport inhibitors (NPA or TIBA). The results show that NPA aggravated Cd-induced root growth inhibition and reduced the formation of lateral roots (Figure 4E,F and Figure S3E). In addition, NPA treatment caused obvious curling of the tomato leaves (Figure 4E). Unexpectedly, low doses (0.5 and 1 μM) of NPA mitigated Cd-induced hypocotyl growth inhibition (Figure S2E). Similarly, TIBA aggravated root growth inhibition caused by CdCl_2_ treatment (Figure 4G,H). However, the number of lateral roots was not affected by TIBA under Cd stress (Figure 4G and Figure S3F). TIBA had no obvious impact on Cd-induced hypocotyl growth inhibition (Figure S2F). These findings imply that maintaining the growth of tomato seedling roots under Cd stress requires the participation of PAT.

### 3.4. Yucasin Reduces Cd-Induced Auxin Accumulation in Tomato Seedling Roots

**Figure 5:**
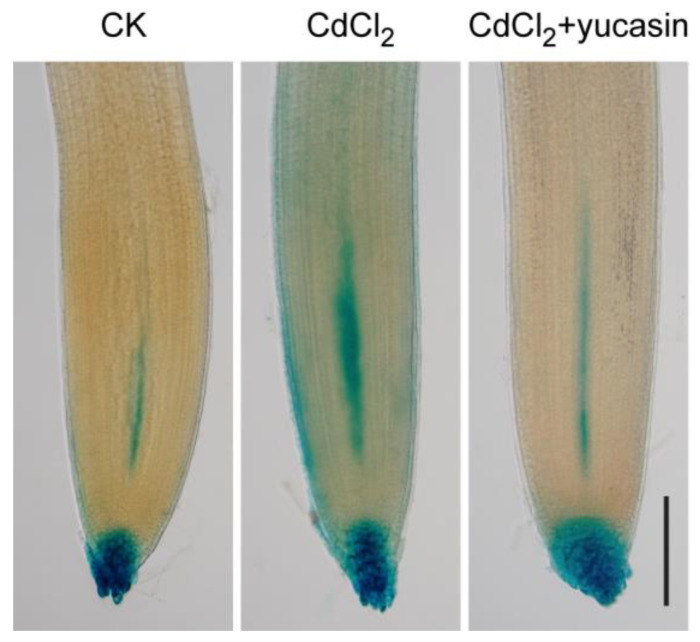
Yucasin reduces Cd-induced auxin accumulation in tomato roots. Analysis of DR5:GUS expression in tomato seedling roots with GUS staining after treatment with 25 μM CdCl_2_ for one hour or 25 μM CdCl_2_ with 10 μM yucasin for one hour after pretreatment with 10 μM yucasin for six hours. Scale bar = 200 μm.

Our experiments suggest that CdCl_2_ treatment leads to auxin accumulation in tomato root tips and that yucasin application can effectively alleviate Cd-induced primary root elongation inhibition. To investigate the role of yucasin in regulating root growth under Cd stress through its impact on auxin response, we examined changes in auxin levels in tomato root tips co-treated with yucasin and Cd. Yucasin significantly reduced Cd stress-induced auxin accumulation in tomato roots (Figure 5) and alleviated Cd-induced root growth inhibition by reducing auxin accumulation.

### 3.5. Auxin Affects Cd Accumulation and Transport in Tomato Seedlings

**Figure 6:**
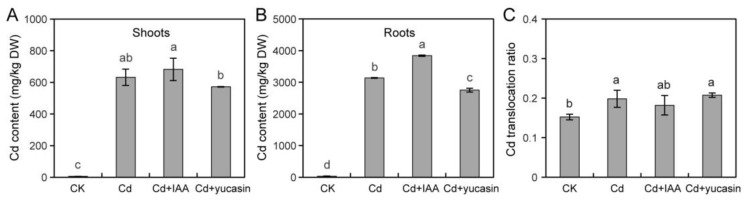
Effects of auxin on Cd accumulation and transport in tomato seedlings. (**A**–**C**) Five-day-old tomato seedlings were subjected to 25 μM CdCl_2_, co-treated with 25 μM CdCl_2_ and 10 nM IAA, or 25 μM CdCl_2_ and 5 μM yucasin for four days. The shoot and root of tomato seedlings were collected and analyzed for Cd content (**A**,**B**) and root-to-shoot Cd translocation ratios (**C**). Values represent the averages ± SD (*n* = 3, Duncan’s test).

Cd is absorbed by roots and accumulates in plants, damaging plant cells and inhibiting plant growth [7]. To evaluate the role of auxin, we explored its impacts on Cd absorption and accumulation in plants by applying IAA and yucasin (Figure 6). Our results show that IAA promoted Cd accumulation in tomato roots, whereas yucasin reduced Cd accumulation (Figure 6B). IAA and yucasin had no effect on Cd accumulation in aboveground tissues, that is, the shoots and leaves (Figure 6A). In addition, Cd transport within plants was not significantly affected by IAA or yucasin treatment (Figure 6C). Consistent with the mitigation of Cd stress-caused primary root elongation inhibition by yucasin, yucasin-treated seedlings showed reduced Cd accumulation in the roots, implying that they experienced low-dose Cd toxicity. These results indicate that yucasin attenuated Cd toxicity in tomato seedlings by reducing Cd accumulation in the roots.

### 3.6. Yucasin Reduces Cd Stress-Induced ROS Accumulation and Root Cell Death

**Figure 7:**
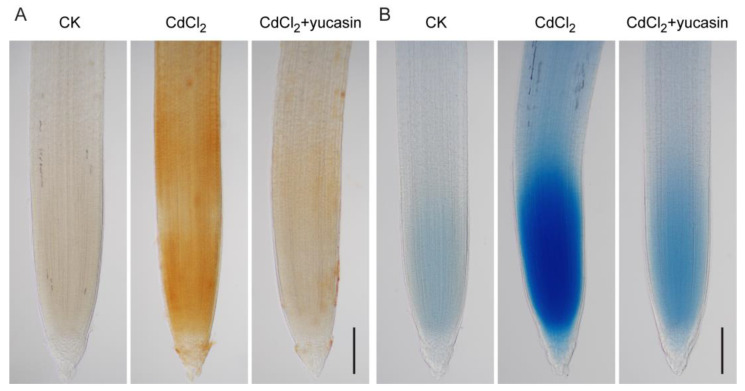
Yucasin reduces ROS accumulation and cell death caused by Cd stress in tomato seedling roots. (**A**). Analysis of ROS accumulation in tomato seedling roots with DAB staining after treatment with 25 μM CdCl_2_ for two hours, or 25 μM CdCl_2_ with 10 μM yucasin for two hours after pretreatment with 10 μM yucasin for six hours. (**B**). Cell death detection in tomato seedling roots with trypan blue staining after treatment with 25 μM CdCl_2_ for twelve hours, or 25 μM CdCl_2_ with 10 μM yucasin for two hours after pretreatment with 10 μM yucasin for six hours. Scale bar = 200 μm.

ROS are important signaling molecules in plant responses to Cd toxicity [12]. Excessive ROS accumulation can cause oxidative damage in plants and trigger cell death. To analyze the mechanism by which yucasin mitigates Cd toxicity in tomato seedlings, yucasin was employed to explore its regulatory role in ROS accumulation and cell death induced by Cd stress (Figure 7). ROS and cell death were detected using DAB and trypan blue staining, respectively. The results show pronounced ROS accumulation and cell death in tomato seedling roots after Cd stress, which was ameliorated by yucasin application (Figure 7A,B), thereby alleviating Cd toxicity in seedlings. This is consistent with the reduction of Cd accumulation and mitigation of Cd toxicity in roots by yucasin application.

### 3.7. Yucasin Mitigates the Impacts of Cd Toxicity on Photosynthetic Pigment Content

To further evaluate the potential contribution of yucasin in mitigating Cd toxicity in plants, we measured the leaf photosynthetic pigment content (Figure S5). Seedlings exposed to CdCl_2_ treatment displayed markedly reduced chlorophyll a, b, and carotenoid contents compared with untreated plants (Figure S5). However, yucasin application markedly elevated chlorophyll a, carotenoid, and total photosynthetic pigment contents under Cd stress (Figure S5). These findings suggest that yucasin mitigates Cd toxicity in seedlings by weakening the effects of Cd stress on photosynthetic pigments.

## 4. Discussion

Among heavy metal soil pollutants, Cd has the greatest impact on plant growth and food safety [1]. This study shows that tomato seedling growth inhibition was caused by Cd stress. Recent studies suggest that applying an auxin synthesis inhibitor, 4-phenoxyphenylboronic acid (PPBo), can effectively alleviate Cd-induced barley root elongation inhibition [32]. Consistently, we observed that yucasin stimulated tomato primary root growth under CdCl_2_ treatment (Figure 4C,D and Figure S4). Plant survival depends on root growth and development, which are crucial for water and mineral ion acquisition. Therefore, exploring the growth, development, and response mechanisms of tomato roots during Cd stress helps reveal plant adaptation mechanisms to stressors. Nonetheless, the potential regulatory mechanism of yucasin involved in tomato root growth under Cd stress still needs to be elucidated.

Auxin is considered an important regulatory factor in plant responses to Cd toxicity [9,12]. However, there are conflicting reports on auxin responses in plant roots under Cd stress. Studies suggest that CdCl_2_ treatment significantly increases auxin accumulation in Arabidopsis [33] and barley roots [32]. In contrast, Hu et al. [9] reported decreased auxin levels in Arabidopsis roots caused by Cd stress. However, Hu et al. [9] also demonstrated that DR5:GUS expression in Arabidopsis roots was enhanced after 12 h of Cd stress treatment and was significantly weakened at 24 and 48 h. Consistent with this, a dynamic alteration of the auxin response signal was observed in tomato roots after CdCl_2_ treatment, characterized by an initial increase followed by a subsequent decrease (Figure 2B). These results indicate that auxin levels change dynamically during Cd stress. Thus, seemingly contradictory phenomena may be caused by different periods of auxin monitoring or by differences in plant species.

Plant root growth under Cd stress is strictly regulated by auxins [12]. Studies show that CdCl_2_ treatment induces auxin biosynthesis and accumulation in rice roots but reduces its accumulation in Arabidopsis roots [19,21]. NAA treatment reduces Cd fixation and accumulation in rice roots; however, it enhances the same in Arabidopsis roots, ultimately mitigating Cd toxicity [19,21]. Here, it was found that exogenous auxin (IAA) treatment increased Cd accumulation in tomato seedling roots (Figure 6B). In addition, NAA treatment aggravated Cd-induced primary root inhibition in tomato seedling roots (Figure 3C,D and Figure S4). These findings suggest that the regulatory mechanisms governing auxin-mediated responses to Cd toxicity vary significantly between species. In contrast, yucasin application reduced Cd accumulation and significantly promoted primary root elongation (Figure 4C,D and Figure 6B). In this study, we observed that the application of 2.5–20 μM yucasin could mitigate the inhibition of tomato growth induced by CdCl_2_ (Figure 4C,D). Specifically, we found that 20 μM yucasin had a strongly promoting effect on tomato primary root growth under Cd stress (Figure 4C,D). This suggests that yucasin holds potential for addressing crop cultivation challenges in Cd-contaminated soils.

Previous studies have shown that auxin interacts with ROS to regulate plant responses to Cd stress [12,34]. Our results suggest that yucasin can alleviate Cd-induced oxidative stress via scavenging ROS (Figure 7A). Consistent with this, another auxin synthesis inhibitor, PPBo, also alleviated Cd toxicity and growth inhibition in barley roots by inhibiting auxin accumulation as well as H_2_O_2_ and NO production induced by mild and moderate Cd stress [32]. These findings indicate that auxin plays crucial roles in mediating plant responses to Cd toxicity by regulating ROS signaling. However, this is contrary to the observations of Wang et al. [12], who proposed that rice lateral root growth was enhanced in stress-free areas through the ROS-auxin signaling pathway and reshaped the root system architecture to avoid Cd stress. This study shows that NAA promotes lateral root development but aggravates Cd-induced primary root growth inhibition (Figure 3C,D, Figures S3A and S4). Yucasin stimulated primary root growth but inhibited lateral root development under CdCl_2_ treatment (Figure 4C,D, Figures S3D and S4). These results suggest that auxins play different roles in regulating the growth of tomato primary and lateral roots under Cd stress.

Collectively, our results suggest that Cd disturbs auxin homeostasis, ROS accumulation, and cell death in tomato roots, resulting in inhibited root growth. Yucasin application not only reduced Cd absorption and accumulation by roots but also reduced ROS accumulation and cell death, thereby alleviating Cd toxicity in tomato seedlings. Additionally, the decrease in photosynthetic pigment contents caused by Cd stress was alleviated in the presence of yucasin. Thus, yucasin is expected to enhance plant adaptability to Cd-contaminated soil, reduce Cd^2+^ accumulation in crops, and ensure agricultural production and human food security.

## Data Availability

The experimental data are presented in the article and Supplementary Information.

## Supplementary Materials

The following supporting information can be downloaded at: https://www.mdpi.com/article/10.3390/toxics12050374/s1, Figure S1. Tomato seedling growth and lateral root development under CdCl_2_ treatment. Figure S2. Effect of auxins or auxin inhibitors on tomato seedling hypocotyl elongation under CdCl_2_ treatment. Figure S3. Impact of auxins or auxin inhibitors on tomato lateral root density under CdCl_2_ treatment. Figure S4. Yucasin stimulates tomato primary root growth under CdCl_2_ treatment. Figure S5. Yucasin attenuates the decrease in photosynthetic pigment contents induced by CdCl_2_ treatment.
