# Supplementary material for: Effect of Auxin on Cadmium Toxicity-Induced Growth Inhibition in Solanum lycopersicum
Source: Toxics. 2024 May 19;12(5):374. doi: 10.3390/toxics12050374 (PMC11125773; doi:10.3390/toxics12050374)
Supplement: Supplementary file 1 [file toxics-12-00374-s001.zip › toxics-2980076-supplementary.pdf]

toxics

MDPI

Supplementary

Effect of Auxin on Cadmium Toxicity-Induced Growth Inhibition in *Solanum lycopersicum*

Huabin Liu [1], Yue Wu [1], Jiahui Cai [1], Yuting Chen [2], Cheng Zhou [1], Cece Qiao [1], Yuliang Wang [1,*] and Song Wang [1,*]

[1] College of Life and Health Sciences, Anhui Science and Technology University, Chuzhou 233100, China; liuhuabin@mail.nankai.edu.cn (H.L.); wy16605662883@126.com (Y.W.); caijh@ahstu.edu.cn (J.C.); zhoucheng@njau.edu.cn (C.Z.); 15150535579@163.com (C.Q.)

[2] College of Life Sciences, Nanjing Agricultural University, Nanjing 210095, China; 2023816122@stu.njau.edu.cn

* Correspondence: roystonea@163.com (Y.W.); wangs@ahstu.edu.cn (S.W.)

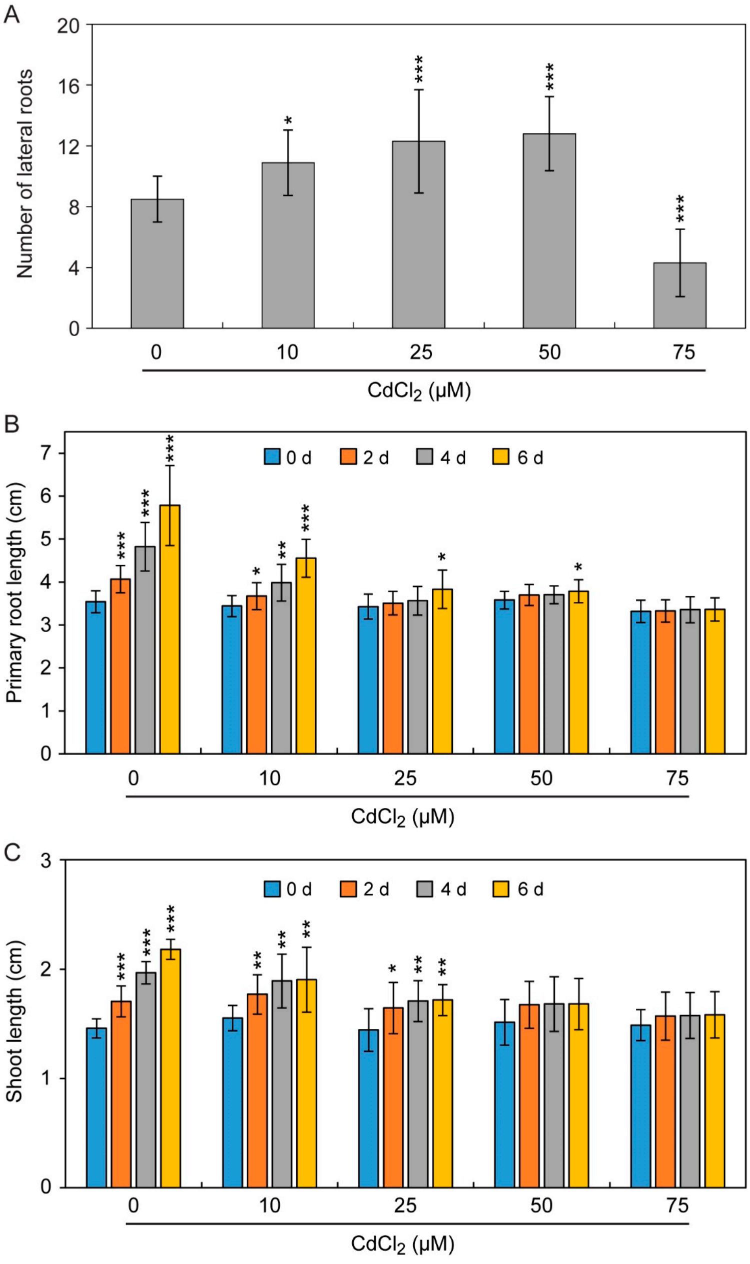

Figure S1. Tomato seedling growth and lateral root development under $CdCl_2$ treatment.

Citation: Liu, H.; Wu, Y.; Cai, J.; Chen, Y.; Zhou, C.; Qiao, C.; Wang, Y.; Wang, S. Effect of Auxin on Cadmium Toxicity-Induced Growth Inhibition in *Solanum lycopersicum*. *Toxics* **2024**, *12*, 374. https://doi.org/10.3390/toxics12050374

Academic Editor: Luis Alberto Henríquez-Hernández

Received: 9 April 2024
Revised: 15 May 2024
Accepted: 16 May 2024
Published: 19 May 2024

Copyright: © 2024 by the authors. Licensee MDPI, Basel, Switzerland. This article is an open access article distributed under the terms and conditions of the Creative Commons Attribution (CC BY) license (https://creativecommons.org/licenses/by/4.0/).

A-C. Five-day-old tomato seedlings were exposed to culture solutions with different concentrations (10, 25, 50, 75 μM) of $CdCl_2$ for six days. The number of lateral roots per seedling was calculated (A). Photographs were taken every two days, and the length of the primary root (B) and hypocotyl (C) were measured by ImageJ software. Values represent the averages ± SD ($n \geq 8$), Student's t-test, $^{*}P < 0.05$, $^{**}P < 0.01$, $^{***}P < 0.001$.

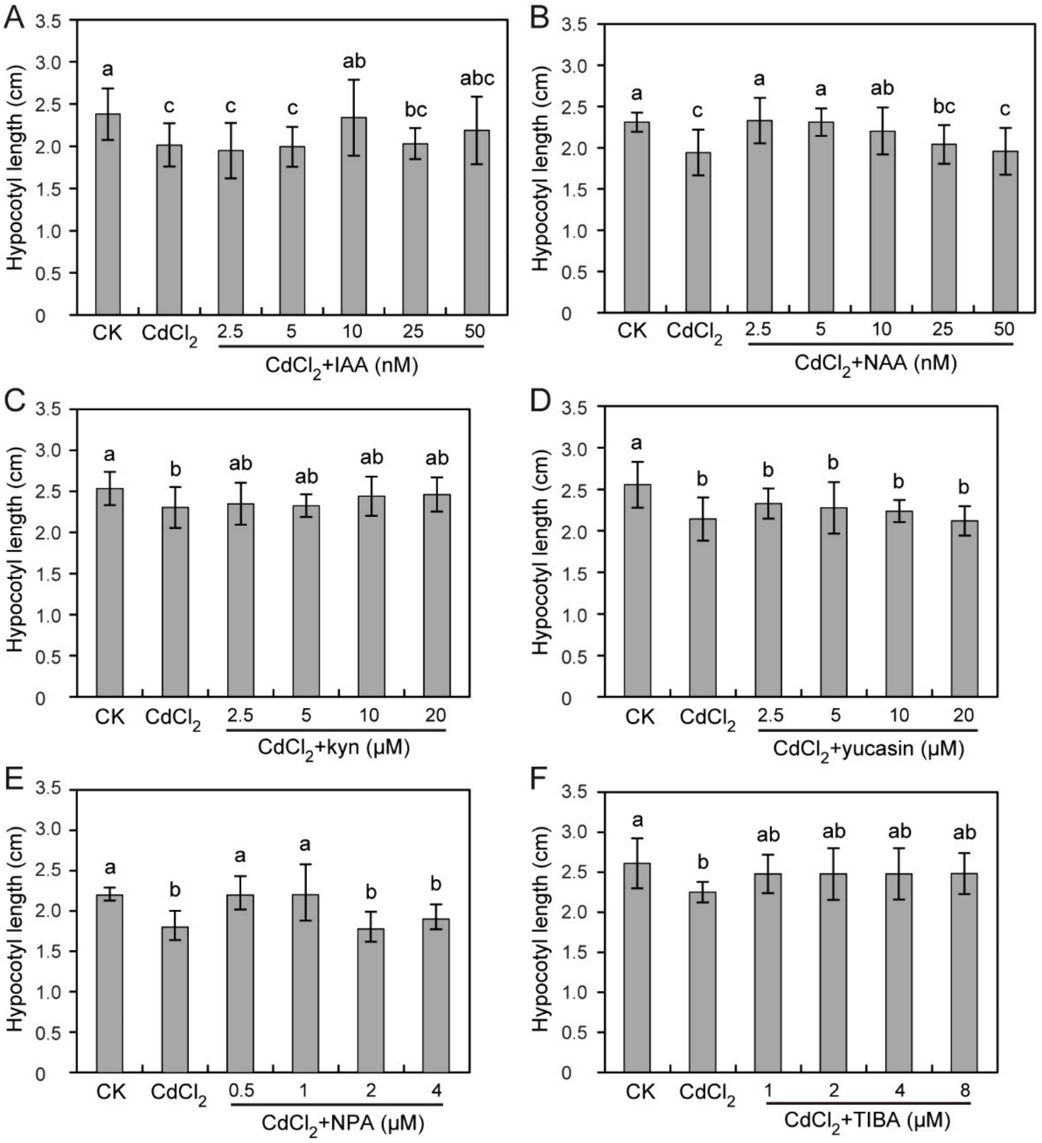

Figure S2. Effect of auxins or auxin inhibitors on tomato seedling hypocotyl elongation under $CdCl_2$ treatment.

A-F. Effect of exogenous auxins (IAA or NAA), auxin biosynthesis inhibitors (kyn and yucasin), or auxin transport inhibitors (NPA and TIBA) on tomato hypocotyl growth under Cd stress. Five-day-old tomato seedlings were subjected to 25 μM $CdCl_2$, or 25 μM $CdCl_2$ with 2.5–50 nM IAA (A) or NAA (B), 2.5–20 μM kyn (C) or yucasin (D), 0.5–4 μM NPA (E), or 1–8 μM TIBA (F) for six days. Values represent the averages ± SD ($n \geq 8$, Duncan's test).

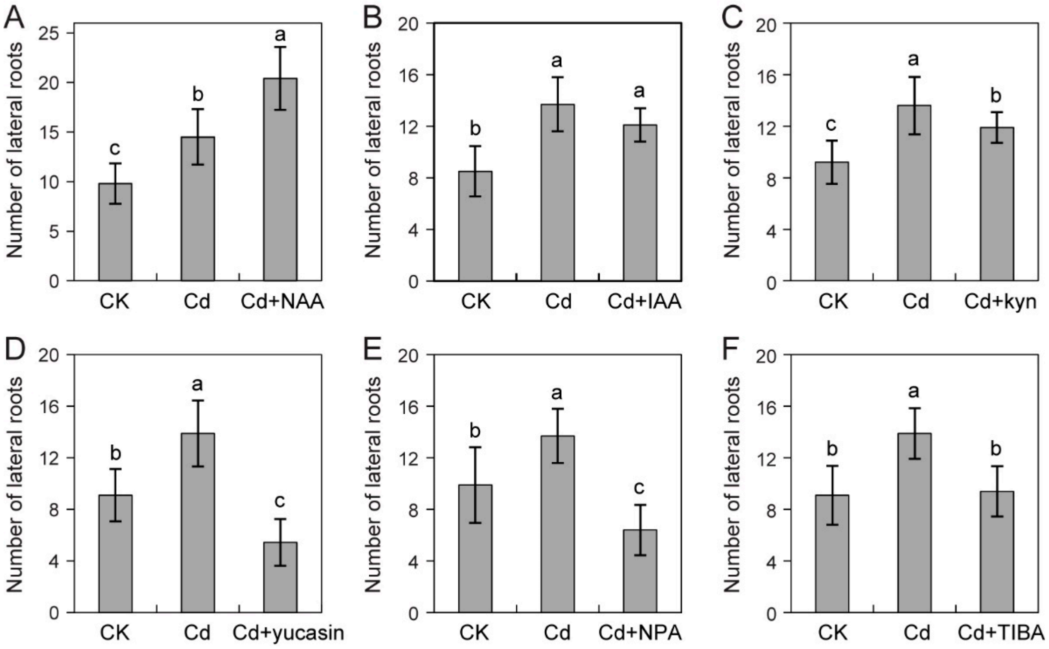

Figure S3. Impact of auxins or auxin inhibitors on tomato lateral root density under $CdCl_2$ treatment.

The number of lateral roots per seedling was calculated. Five-day-old seedlings were subjected to 25 μM $CdCl_2$ or 25 μM $CdCl_2$ with 50 nM NAA (A), 50 nM IAA (B), 20 μM kyn (C), 20 μM yucasin (D), 4 μM NPA (E), or 8 μM TIBA (F) for six days. Values represent the averages ± SD ($n \geq 8$, Duncan's test).

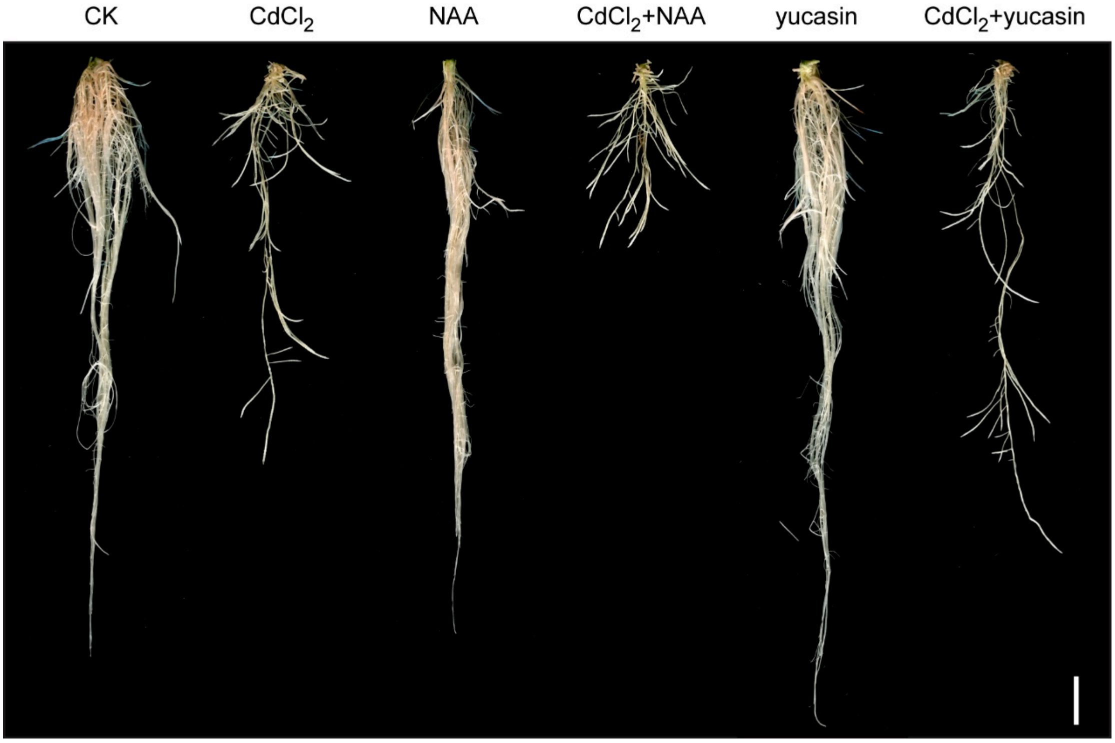

Figure S4. Yucasin stimulates tomato primary root growth under $CdCl_2$ treatment.

Five-day-old tomato seedlings were subjected to 15 μM $CdCl_2$ or 15 μM $CdCl_2$ with 2.5 nM NAA (A, B) or 1 μM yucasin (C, D) for three weeks. The solution was refreshed every three days.

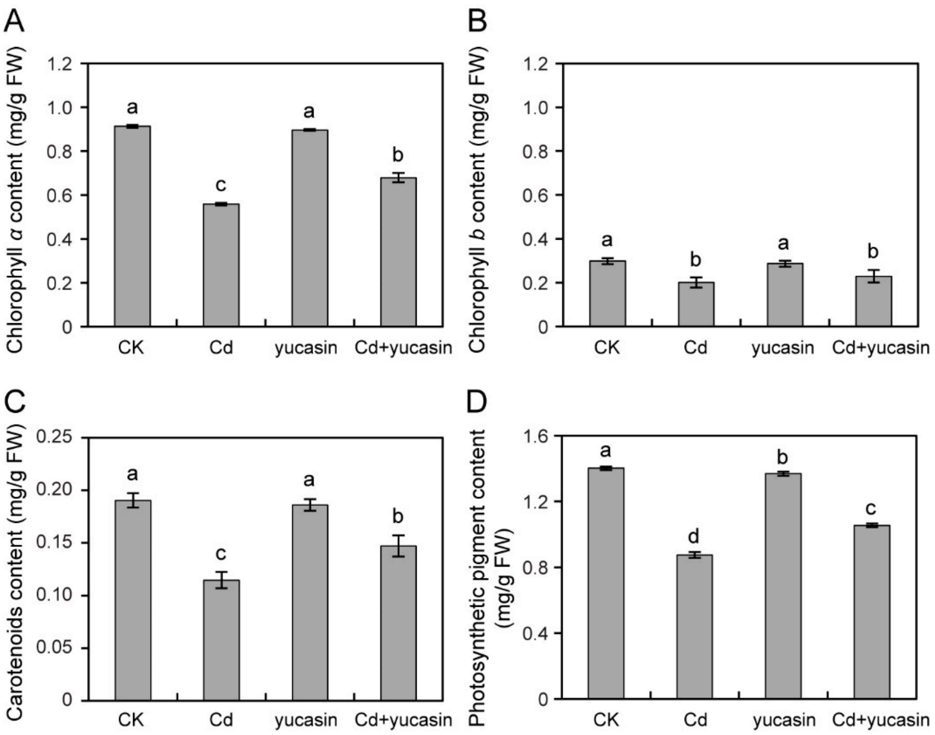

Figure S5. Yucasin attenuates the decrease in photosynthetic pigment contents induced by $CdCl_2$ treatment.

A-D Five-day-old tomato seedlings were treated without or with 25 μM $CdCl_2$, 2.5 μM yucasin, and both 25 μM $CdCl_2$ and 2.5 μM yucasin for four days. Chlorophyll a (A), b (B), carotenoid (C), and total pigment content (D). Data represent the averages ± SD.

Disclaimer/Publisher's Note: The statements, opinions and data contained in all publications are solely those of the individual author(s) and contributor(s) and not of MDPI and/or the editor(s). MDPI and/or the editor(s) disclaim responsibility for any injury to people or property resulting from any ideas, methods, instructions or products referred to in the content.
